# How Can We Engage Oncology Care Providers and Glioblastoma Patients in Conversations About Physical Activity: A Qualitative Descriptive Study Using the Theoretical Domains Framework

**DOI:** 10.3390/curroncol32040197

**Published:** 2025-03-27

**Authors:** Jodi E. Langley, Grace Warner, Christine Cassidy, Robin Urquhart, Mary MacNeil, Melanie R. Keats

**Affiliations:** 1 Faculty of Health, Dalhousie University, Halifax, NS B3H 4R2, Canada; grace.warner@dal.ca (G.W.); ccassidy@dal.ca (C.C.); melanie.keats@dal.ca (M.R.K.); 2 Beatrice Hunter Cancer Research Institute, Halifax, NS B3H 0A2, Canada; robin.urquhart@dal.ca; 3 Faculty of Medicine, Dalhousie University, Halifax, NS B3H 4R2, Canada; 4 Division of Medical Oncology, Nova Scotia Health, Halifax, NS B3S 0H6, Canada

**Keywords:** cancer, physical activity, glioblastoma, theoretical domains framework, healthcare communication

## Abstract

Glioblastoma (GB) is the most common primary malignant brain tumour in adults. Physical activity (PA) has value as a supportive service for individuals living with a GB diagnosis to help maintain quality of life and physical functioning. The objective of this study is to understand how oncology care providers (OCPs), family/friend caregivers, and health system decision makers can include conversations of PA into care for those living with a GB. We conducted 19 semi-structured interviews guided by the Capability, Opportunity, Motivation–Behaviour (COM-B) model and further refined them by the theoretical domains framework (TDF). The data were then analyzed using a directed content analysis using a codebook generated using the TDF. Patients and family/friend caregivers appreciated hearing about PA from their OCPs, from initial diagnosis into follow-up appointments, and they saw PA as a way to take a break from cancer/medically focused care, and historical PA behaviours did not mean patients were more or less likely to be open about PA discussions. This study further emphasises the inclusion of PA discussions in clinical care. OCPs in GB care feel they have the knowledge to partake in PA conversations, and GB patients are open to having these conversations. However, specific barriers are in place that do not lead to widespread implementation of PA discussions for all patients.

## 1. Introduction

It is estimated that in 2023, 3200 Canadians were diagnosed with brain and spinal cord cancer, with as many as 2500 Canadians dying from the disease [1]. Glioblastoma (GB) is associated with significant morbidity and high symptom burden (e.g., seizures, fatigue, motor deficits, cognitive decline, and poor quality of life) [2]. Despite the poor prognosis and high symptom burden, there is an increasing awareness that physical activity (PA) can lessen these symptoms and offer a way for patients to maintain or improve their quality of life and physical function [3]. PA has value when considering the care for individuals living with a GB diagnosis and should be considered a supportive service in their care. Individuals living with and beyond cancer do not engage in PA comparatively to their healthy counterparts [4], and depending on their tumour type, stage of disease progression, treatments, and supports, they can be even more inactive [5]. It is important that people living with GBs have support to engage in PA, particularly from their oncology care providers (OCPs).

OCPs serve as an important source of motivation for individuals along their cancer journey and are critical in encouraging patients to lead a healthy lifestyle. OCPs often have a longitudinal relationship with patients, which puts them in a good position to engage in PA discussions at different timepoints throughout diagnosis and treatment. OCPs see patients on a regular basis and are aware of potential limitations and symptom burden. This places OCPs in a unique position to offer brief, tailored PA discussions. However, there are a limited number of OCPs that discuss PA with their patients [6] Approximately 60% of cancer survivors have reported that exercise was not discussed with their oncologist [7]. There is a lack of research on how OCPs can engage in conversations about PA with individuals living with a GB. Individuals living with and beyond breast cancer are receptive to discussing PA with their oncologist, and when the OCP recommends exercise, there is short-term increase in exercise behaviour [8].

There are many barriers to discussing PA in a clinical setting, including limited knowledge of exercise guidelines, time, limited referral pathways, and a lack of readiness to discuss PA [9]. PA counselling is often complex, and distressing symptoms can cause patients to not partake in PA [10]. Despite these behaviours, there is clear evidence that PA can improve symptom management at any point on the cancer care continuum for all cancer types [11,12]. Healthcare providers who do give PA advice typically offer casual advice (e.g., walk more) and do not provide specific advice (e.g., try and go for a 10 min walk 3x/week), leading to lower long-term PA adherence [13]. Of note is the fact that these initial interaction with patients can be seen as ‘teachable moments’ that can kickstart new health behaviours [14]. There is limited uptake of PA discussion in a clinical setting; this is unfortunate given the extensive research indicating that PA is beneficial for those living with and beyond cancer [15,16].

Within cancer care, there is a need to explore ways in which OCPs can have effective, focused clinical visits with patients. When trying to find ways to implement a new intervention into standard of care (i.e., formally adding PA discussion into clinical visits), implementation researchers recommend using a theory-based approach to identify barriers and facilitators to the target behaviour. The Behaviour Change Wheel (BCW) is a theoretical framework that can be used by researchers to increase the likeliness that interventions are implemented effectively and are sustainable by using its components, including the COM-B model and Theoretical Domains Framework (TDF) [17,18]. This is of particular interest to facilitate OCP and patient discussions regarding PA. A theory-based approach provides clear definitions surrounding the parameters of this behaviour and, if needed, direction on what tools can be designed. The BCW COM-B model and the TDF have previously been used to develop an understanding of the use of PA guidelines within oncology to help inform and develop a more robust understanding of translating exercise guidelines into practice [19].

This study builds on an existing body of literature demonstrating that PA is beneficial for people living with and beyond cancer [14]. Preliminary studies are showing a positive response to tailored PA [19,20], and the patient–clinician relationship is key; therefore, when an OCP provides PA advice in an empathetic and educated way, it is likely to have positive results on their patient. There is a need to further explore the evidence gap of why PA is not formally discussed or recommended in clinical settings. Barriers to this behaviour within a complex system are important to consider when designing interventions to improve clinical discussions around PA. Therefore, the aim of this study was to understand the barriers and facilitators that OCPs, family/friend caregivers, and health system decision makers (e.g., middle managers, senior leadership) have when it comes to PA and PA discussion in clinical care. Further, the aim was to gather perspectives on how these conversation are currently happening for those living with a GB diagnosis. The main research question guiding this study was as follows: how are clinicians currently engaging in PA discussion in a clinical setting with GB patients? The sub-questions were as follows: (1) How do GB patients feel about discussing PA with their OCPs? And (2) how can we best support OCPs in engaging in PA discussions in clinical context?

## 2. Methods

### 2.1. Study Design

This study is part of a larger study being conducted to develop multiple theory-informed interventions to improve PA discussions in clinical care for those living with non-curative cancer. The context of the study was health services in Nova Scotia, Canada. The Canadian government funds the Nova Scotia Department of Health and Wellness (sets public health policy) and Nova Scotia Health (provides health services and programs) to deliver oncology services through individual health professionals. A qualitative descriptive design was used in this study [21,22] to conduct semi-structured interviews with patients, OCPs, family/friend caregivers, and health decision makers. This study was approved by Nova Scotia Health Research Ethics Board (REB# 1028770).

### 2.2. Theoretical Approach

The BCW is a systematic, theory-based guide to intervention design based on the principles of the Capability, Opportunity, Motivation–Behaviour (COM-B) model. The BCW is a synthesis of 19 existing behaviour change frameworks that offers a comprehensive and systematic guide to intervention design [17]. The BCW includes an analysis of the nature of the behaviour, the mechanisms that need to be addressed in order to create behaviour change, and the interventions and policies required to change those mechanisms [17]. The Theoretical Domains Framework (TDF) is a variant of the COM-B model that further categorises behaviours into barriers and facilitators to find specific factors that influence the COM-B model [17]. The TDF allows for the framing of how certain factors (e.g., PA behaviour) affect key behaviours such as social influences, emotion, reinforcement, behavioural regulation, physical skills, etc. [18]. It allows for a better understanding of how and when OCPs discuss PA with their patients. In this study, the TDF was used with the BCW to aid in (i) understanding the perspective of participants on PA discussion within a healthcare setting for those living with a GB diagnosis; and (ii) how care teams and health system decision makers can support the use of conversations of PA into clinical visits. See Appendix A for a breakdown of the TDF and a description of each domain in relation to this study.

### 2.3. Participants/Recruitment

Participants were recruited through a variety of methods from all areas of Nova Scotia. Patients and family/friend caregivers were given the research team’s contact information when they were at the Nova Scotia Cancer Center by their care team and contacted the research team directly if they wanted to participate. Several patients were previous participants in a clinical trial from one of the senior authors’ (MK) labs. OCPs were recruited through their work e-mail, or they were given the lead authors e-mail to contact when it was convenient. Purposive sampling [23] was used with OCPs and health decision makers by targeting multiple professionals’ roles within the Nova Scotia Healthcare system to investigate a broad range of perspectives and patterns that exist within the healthcare system. These targeted individuals included oncologists, oncology nurses, social workers, surgeons, and other allied health professionals. The healthcare decision makers included those who make decisions regarding healthcare initiatives in Nova Scotia. This included leaders in the cancer care program, middle managers, programme directors, and other key individuals. After each interview, participants were asked if anyone else could be interviewed, and these individuals were contacted directly by the research team (i.e., snowball sampling).

### 2.4. Inclusion and Exclusion Criteria

Participants were informed of the methods and study design verbally and in writing before providing verbal informed consent. Participants were screened by the primary author and had to meet the given inclusion criteria. Participants were remunerated for their time (patients and family/friend caregivers: CAD 35 per interview; health decision makers and OCPs: CAD 25 per interview). All participants had to meet the following criteria: reside in Nova Scotia, be >18 years of age, and be able to speak and write in English (to provide informed consent). Specific inclusion criteria were as follows: patients diagnosed with a GB and who have a predicted life expectancy of >3 months; family/friend caregivers currently caring for someone who has been diagnosed with a GB; and oncology care providers and health decision makers currently or previously caring for patients with a GB diagnosis or who oversee programs for people living with a GB and who work for Nova Scotia Health or Department of Health and Wellness or have a role in providing formal support.

### 2.5. Data Collection

A semi-structured interview guide based on the BCW-COM-B and TDF was created [19] that probed on beliefs about PA discussion in clinical behaviour, including barriers and facilitators to having these discussion and barriers and facilitators to engaging in PA with a GB diagnosis. The 4 semi-structured interview guides (patients, family/friend caregivers, OCPs, and health decision makers) are provided in Supplementary File S1. The semi-structured guide enabled flexibility so that the interviews could explore certain issues in greater depth. Interviews were conducted between June 2023 and January 2024. OCPs and health decision makers were interviewed first, followed by patients and family/friend caregivers. Interview questions probed participant narratives to understand factors that affected OCP and patients’ abilities to engage in PA conversations during clinical visits. Interview questions covered perceptions of PA, organsational and health system determinants, barriers and facilitators to PA conversations, and details of what occurs during appointments. Open discussion was encouraged in order to identify and explore evolving themes. The primary author (JL) conducted all interviews over zoom, phone, or in person. Interviews ranged in length, with a range of 64–128 min, and the average interview length lasted 74 min. All interviews were audio-recorded and transcribed verbatim by an external transcriber. After preliminary interviews were completed and data were coded and analyzed, 4 additional repeat interviews were conducted from each participant group to probe the legitimacy of the themes.

### 2.6. Data Analysis

Semi-structured interviews were read and re-read by the lead author (JL) to develop a working understanding of what happens during clinical visits, how individuals feel about PA discussion, and barriers/facilitators to not only the discussion around PA but also PA behaviour and other disease-specific factors. The data were then analyzed using a directed content analysis [24] using a codebook generated using the 14 TDF domains (see Supplementary File S1). First, the transcripts were read by the lead author (JL), who then categorised elements in accordance with the COM-B model and then grouped them into the 14 TDFs. Secondly, after discussion with senior authors (GW and CC), these were further refined. Similar themes were grouped together to create subcategories based on the TDF domains. Following this, an inductive coding took place to generate subcategories of similar beliefs surrounding the target behaviour (discussing PA with patients in clinical settings) [25]. Themes were then inductively generated by grouping subcategories around the themes within the OCP interviews. Then, patients, family/friend caregivers, and health decision makers interviews were used to refine working ideas and understand structural and institutional factors that hindered and/or facilitated having PA discussions in a clinical context. Following this, the themes were sent to all authors for review, during which clarification of the themes was discussed. Further, 4 follow-up interviews were used to refine the themes and test the current themes within each sub-group.

## 3. Results

Nineteen individual interviews were conducted with GB patients (n = 5), family/friend caregivers (n = 2), OCPs (n = 8), and health decision makers (n = 4), with four additional follow-up interviews (one individual from each sub-group). Given the diverse sample of participants and the unique perspectives described by each group, themes were individually created for each group of participants. GB patients and their family/friend caregivers were grouped together, as they reported similar experiences when discussing the target behaviour of discussing PA in clinical settings. OCP interviews were conducted with two medical oncologists, two radiation oncologists, three oncology nurses, and one surgeon. This represents a majority of the staff that work with GBM patients, with only two surgeons turning down interviews due to lack of time. The views of a palliative care physician and nurse are not represented, as they were unable to be contacted for an interview. Seven of the eight OCPs identified as Caucasian, with five identifying as women. A total of five patients were interviewed, with two identifying as women (three identifying as men), and all five described themselves as Caucasian. All four health decision makers identified as women, with three identifying as Caucasian. Interestingly, only two patients resided in an urban centre, although all the OCPs and health decision makers resided in an urban centre.

### 3.1. Summary of Themes

Table 1 presents an overview of the themes from the interviews that were mapped onto the BCW, incorporating the COM-B model and the TDF. The findings highlighted to some degree the use of all 14 TDFs in the data, with cross-referencing across all COM-B model components. Most themes could be allotted to seven different reoccurring themes, as seen below. The six TDF domains were highlighted due to their consistency across interviews and perspectives.

#### 3.1.1. Category (1): Patients and Family/Friend Caregivers

Theme (1): Patients appreciated hearing about PA from their OCPs, from initial diagnosis into follow-up appointments.

Patients discussed how they trust their providers’ education and training, and this relationship led them to think about PA in a different way. One participant shared the following:

*“So smart people say, ‘Hey, exercise helps you get a little bit longer and a little bit further along’… why wouldn’t I?”*

GB 10 (patient)

Patients liked talking about their PA journey with their OCPs throughout their treatment. OCPs offered support and acted as a motivator for patients; this was reflected by patients when they talked about the help and assistance from their OCPs. As illustrated below, one patient discussed ways in which they valued their relationship with their OCP to give that extra boost for exercise:

*“It’s the exercise that have helped me the most. We’ve {OCP and patient} been really sort of adamant about that. Because even when I go in and I’m not feeling well, if I exercise, regardless of the level of exercise I can do, I always feel better afterwards.”*

GB11 (patient)

Theme (2): PA can aid as a break from cancer/medically focused care.

When asked if patients found it hard to talk about PA in their initial appointment, patients described how talking about PA with their OCPs provided “relief” in their appointment compared to the other more distressing aspects of their care. When asked if patients felt it was overwhelming to come in and discuss PA right after their diagnosis, they stated the following:

*“ … for me it was good because we both are very active. And it’s… We want to continue being active. So for us it was nice and refreshing, as opposed to all the doom and gloom. It was somewhat comfortable. So it was good.”*

GB 08 (family/friend caregiver)

Patients expressed feelings of discouragement and could feel overwhelmed. They go through a lot of medical treatment, side effects from treatments, and emotional distress. One patient discussed how they have lost a lot of independence:

*“[tearful] I don’t get to teach anymore. I don’t get to drive. I feel like a lot of doom and gloom sometimes.”*

GB11 (patient)

This same patient (GB11) later discussed how engaging in PA classes that were prompted by their OCP allowed them to be at a place for social support:

*“… I think… is that it’s more than just an exercise program, right? So, you know, [instructor], if it comes to a point where people can’t exercise but they want to come for the social, she encourages that. Like it definitely took a life of its own, right?”*

GB11 (patient)

Patients expressed having a desire to continue to be physically active and were encouraged by talking about it with providers, and they viewed it as something they could control and that was separate from the medical care. One patient discussed the ability to “drive the bus” of PA:

*“The exercise thing is pretty much giving me the only thing that I can hold in my hand. And hold it and say, ‘I have control over this. This bus I drive.’ If you want to do more exercise, different exercise, harder, easier, more or less, I ask some smart people how to do it smartly, and they tell me how to do it smartly. And I do it that way, and I get benefits. It’s the only thing that gives me a positive feel in a morass of you’re in a fog bank running towards a cliff.”*

GB10 (patient)

There was an understanding that PA behaviour will inevitably change, and this was discussed with their OCPs. Family/friend caregivers noted a difference in not being able to carry out PA alone anymore, as they were not able to leave their loved one alone due to seizure risk and how that consequently affected their PA. One patient discussed a change in their own PA behaviours due to the risk of seizures; however, they had adapted to make it work:

*“I love having people around. But yes, I do also enjoy my… [alone time] Like I would go for a two, three hour walk in the woods, and be totally happy with that. But we’ve adapted.”*

GB09 (family/friend caregiver)

Theme (3): Historical PA behaviours did not mean patients were more or less likely to be open about PA discussions.

Having a history of PA may increase the likelihood of being open to PA; however, patients who did not have a history of PA saw OCPs bringing it up as a tipping point to start. One patient expressed they never were physically active prior to their diagnosis but they had changed their mindset:

*“Physical activity wasn’t really a part of my life. Or my wife’s life. It wasn’t necessary for my job. … It just never came to me to do that. Which you would be accurate in saying, ‘Now, [participant] that you’re facing this journey, now you’re leaning into it, and your body is losing weight, and you’re putting on muscle where muscles weren’t really that big before.’ You’re right. Someone might look at that and say, ‘Well, [participant], what changed with you?’ [referring to starting physical activity journey after diagnosis]”*

GB10 (patient)

Individuals with a history of PA were confident about their abilities to continue to be physically active throughout their diagnosis. They relied on their OCPs to provide approval to continue to be physically active and to provide special considerations given their diagnosis. One patient expressed hearing things like “*don’t hold your breathe while exercising*”, and although intuitive, it was nice to hear:

*“What’s really good about that is because as you’re doing exercise and all that, it’s nice to know that. So okay, don’t hold your breath. And even though you think that, it’s nice to hear that.”*

GB08 (family/friend caregiver)

When patients were referred to a structured exercise programme from their providers, it allowed them to feel confident in the exercise that they were carrying out. This also allowed patients with a history of PA to look at PA in a different way:

*“And I mean it’s [online cancer and exercise class] a really excellent way for people like me who… Like in the wintertime when you can’t really… You know, you’re afraid you might slip and things, or things like that, it’s just so wonderful to have it here. And it’s very challenging. Which I liked.”*

GB16 (patient)

#### 3.1.2. Category (2): Oncology Care Providers

Theme (1): OCPs used different types of methods to talk about PA and offer advice that they felt patients could handle.

When OCPs were asked if they mentioned PA in appointments, some clinicians were adamant about discussing PA with their patients. They described the use of “cue words” for when to talk about PA and movement with their patients. Fatigue was mentioned as a cue word for many OCPs, especially given it is so common in patients; other less often used cue words included stiff and tired. One participant said the following:

*“The minute they ask me about fatigue, they’re like, “Oh, I’m so tired all the time.” And this is my statement I say all the time, I say, “You need to listen to your body. If you have a bit of energy, try to do exercise. Try to do something. Go for a walk, get outside, fresh air, whatever. Do something. That’s the best thing you can do.” But on the other hand, if you’re really tired and your body’s telling you need to rest, you need to rest. But I’m like, “You have to listen. You have to be in tune with your body. You need to listen.”*

GB01 (Nurse)

OCPs also noted that they would bring PA up at different timepoints or revisit it at a later date, depending on the patient’s response and PA habits. They emphasised the need to reiterate past advice or offer new advice so that communication was clear.

*“And that’s when I then make that message clear again, if it’s needed. Some people are super keen, and they’re out walking and stuff. So they’re doing fine. But some… And, you know, not everybody takes your advice.”*

GB05 (Oncologist)

Some OCPs noted that they would show patients how to perform exercises to aid in educating them on ways to maintain physical function and activities of daily living.

*“And specifically, I show them how to do the sit to stand, about having a chair and someone… Having it so it won’t move. Putting their hands up on the table, and use that as balance. And try to do it eventually though without, you know, using your arm. So I try to keep them at least with their lower body strong.”*

GB05 (Oncologist)

Some clinicians discussed mentioning PA more casually, with no specific prescription around it. However, they did mention different modes of PA depending on the individual, including light walking to formal research exercise programs. If a patient already had an exercise routine, they discussed having an open discussion about how to adapt this routine to their new body.

*“I guess I would say more as a mentioning it, and I do try to promote it. I try to encourage… But say, you know, if you can do some exercise, and I often give them an example of just going for a walk, light exercise where you might take 10 or 15 min. And if you managed well with that, gradually increase it or go more than once per day… It’s more just as advice. I don’t kind of go into details… For those who do more vigorous exercise, I kind of counsel whether that’s wise. You know, should you cut back?”*

GB07 (Oncologist)

Given the significant distress resulting from a GB diagnosis and treatment, including functional and cognitive decline and poor prognosis, many OCPs expressed that timing of bringing up PA with GB patient was often difficult.

*“But it’s just… Sometimes it’s a bit too much for the patients. They’ve just been diagnosed, and they’re getting this bad news.”*

GB06 (Nurse)

However, some OCPs understood that PA will look very different at each phase of treatment. For example, to minimise post-surgical complications, surgeons will discuss the importance of moving their legs post-surgery as a way to reduce the risk of a pulmonary embolism.

*“And people who have brain tumors and other neurological conditions, they have a higher risk of that [pulmonary embolism] because they’re immobilized on one side or they may be paralyzed on one side. And around the time of surgery, we’re very cautious of that. So one of the things that I always like to ask people to do in bed is to move their legs. Literally move their legs every time they think about it. And I don’t know if you’d call that exercise. It’s pretty… It would be pretty loose definition of exercise. But it’s to help make sure that they are mobile.”*

GB18 (Surgeon)

Theme (2): Formal exercise programs may act as a prompt to have a discussion around PA.

While exercise is not currently standard of care, access to formal exercise support is available through research studies. Providers can directly refer to these programs, and some are champions for these programs and often refer to them directly for patients to enroll in research programs. When formal exercise programs are available, providers who are aware can and do refer to these programs and use them as a prompt to discuss with other clinicians to engage in PA discussions in the clinic. One OCP discussed the use of exercise studies for brain tumour patients as a way to lead into clinical conversations about exercise:

*“And then we do tell them there are some exercise studies being done, you know, specifically for cancer patients and brain tumour patients. And that, you know, we can give them information about the studies or contacts.”*

GB 07 (Oncologist)

Providers mentioned that even when certain PA promotion is targeted towards patients, there can be a trickle-down effect to providers to engage in more PA discussion, and they can see how it fits into the landscape of care:

*“You know, I still think that sometimes, you know, if you had a [exercise] video, you know, things running in the clinic like on the screen. You know, a video. Yeah, but that also is how you get staff, too, to think about it?”*

B05 (Nurse)

OCPs will assist their patient in identifying and choosing what might work best for them. For some patients, that may mean offering encouragement and continuing to be active in their own way, and for others, that may mean referral to an existing exercise program.

*“Fatigue is a huge, huge thing if they go ahead with starting their chemotherapy. So we always think exercise. And we tell them like… You know, we tell them about little [exercise] programs there. Because, you know, if you’re tired, it’s hard to function… Or, we’ll say, ‘Listen, we want you to live life, we want you to go out and about, we want you to exercise.’”*

GB12 (Nurse)

#### 3.1.3. Health Decision Makers

Theme (1): OCPs have a lot of information to relay to patients during appointments, and there is a need to streamline processes

Health decision makers described systematic barriers and overall workload demands that may cause providers to disengage in PA discussion with patients. When referring to PA programs (which can act as a prompt for clinicians to bring up PA), it was noted that there is already a high number of forms that clinicians must fill out during visits, and adding another form may be overwhelming:

*“Yeah, I mean I think some of it was timing in terms of the pandemic was just starting, and people, I think, felt overwhelmed. I think people also just felt, ‘Oh, this is just another tool, another thing.’ We already have three, four, or five forms to complete, whether it’s booking forms for treatment or assessment forms that are already being done.”*

GB 08 (Middle manager)

Noting that OCPs are at the forefront of cancer care, health decision makers highlighted the need to educate OCPs on the importance of PA and empower them to support their patients in being more active:

*“Okay. So the nurse is really the person, and the doctor, that really are the people that are seeing these patients, they are the ones that are in front of them… If they don’t think exercise is important, if they don’t think that diet is important, that stops there… So with that being said, is having that breakthrough of being able to educate, show, teach, enable, the frontline. Because with positive results, it’s like a weed, it grows wild… And it gets to other patients. When patients have a good experience with certain programs, they are your marketers.”*

GB14 (Leadership)

Health decision makers stated the need to support patients and OCPs to have more resources readily available that were tailored to patient concerns, which can lead to a more tailored conversation around what is most helpful for patients given their unique circumstances. Health decision makers expressed the use of self-identification tools being used to help trigger OCPs and patients to have a wider conversation about goals of care and needed support:

*“Give patients the opportunity to self-identify what issues are of concern to them. Have time available to provide some basic information around smoking cessation, around advance care planning, goals of care, conversations around physical activity. Like around some of these important subjects that even if a patient doesn’t raise them, wouldn’t take very long for us to be able to… But I think they get forgotten about. Like they’re not… There’s no triggers. So we need triggers in place that help nurses and other frontline staff remember to have conversations about some of this stuff.”*

GB02 (Leadership)

Health decision makers expressed that they wanted to work with OCPs to find ways to streamline PA resources for patients. One health decision maker expressed the use of clinician champions that have taken this on individually but also the need to streamline this process in order to make this sustainable:

*“And she’s a nurse educator. And so she and I have been in contact a few times. And I think that she’s the one who’s really trying to support sort of embedding that into sort of a systemic pathway so people are getting screened.”*

GB04 (Middle manager)

Health decision makers also expressed that historically, the complexity of healthcare systems has acted as a barrier to implementation; however, they also expressed the critical need for things to change in the current healthcare landscape. One health decision maker was very adamant about this and was actively doing their best to change historical systems.

*“You know, my motto was always like just because this is the way we have done it doesn’t mean this is how we should do it.”*

GB15 (Leadership)

Theme (2): Past programme success can aid in implementation of other programs or models of care.

Health decision makers reflected a lot on programs that have worked in the past and key models of success. One exemplar was a smoking cessation programme, in which nurses were trained to deliver smoking cessation in clinical visits. This was more successful at a smaller cancer centre, where a champion nurse educator took this on and was able to implement it across the cancer centre in a standardised model.

*“They also had some very keen champions who really just took it on. And clearly, if you had nine nurses willing to take extended training to act in that way, that’s what made it happen. So it’s really like the people, the size of the venue, having [champion] there. And they also had a nurse educator who I think played a really big role in facilitating communication across levels of decision-making, as well as across program areas.”*

GB04 (Middle manager)

Further, health decision makers highlighted the use of other palliative care programmes, in which success was seen when working with paramedics. The health decision maker was unsure as to how this success happened but highlighted the importance of reporting and how that can aid in implementation and, in turn, sustainability.

*“Because to be honest, working with the paramedics was the best experience I had. They’re so open. And I feel like… I know there’s a lot of controversy between public and private health care. But, you know, having the paramedics report through to a private organization that’s not Nova Scotia Health, I don’t know what they do differently, but I feel like they do it right.”*

GB02 (Leadership)

## 4. Discussion

In this study, we used the BCW incorporating the COM-B and TDF model to understand the perspectives and behaviours of patients, OCPs, family/friend caregivers, and health decision makers on how clinical conversations of PA are incorporated into care for those living with a GB diagnosis. In our themes, we described the barriers and facilitators to discussing PA in clinical contexts. These findings indicate that patients are open to having conversations about PA in clinical settings and that conversations are already happening in clinical care; however, specific implementation barriers exist that can reduce PA discussions. Overlaying the COM-B model and TDF allowed for a comprehensive theoretical analysis of the participants’ capability, opportunity, and motivation and how these components work together to influence their decision to discuss PA in a clinical setting as a formal supportive service.

### 4.1. Psychological Capability

The findings from this study indicate that clinicians feel they have the capability and knowledge to discuss PA with patients. However, they are focused on primary medical care (e.g., surgery, medications) and rely on the use of prompts to bring up PA. When they are prompted, they do engage in these conversations, often citing patient fatigue as a prompt. This highlights that when discussing PA with patients, not only do (1) OCPs have the knowledge to have these discussions but (2) OCPs also have the communication skills to relay the information to patients. However, they may not have the memory, attention, and decision processes, as OCPs do require prompting. This supports the need for the implementation of more patient-centred communication tools that could act as a prompt. These tools are a proven mechanism for improving outcomes of patients [26,27]. In understanding patient-centred communication, it is important to allow both sides (patient and physician) the opportunity to address specific, relevant concerns, which, in turn, can lead to a more focused discussion [28]. Previous research has also indicated that giving patients a “prompt list” facilitates conversations with patients and physicians [29]. There could be a subset of questions about supportive services for patients. It is noteworthy that clinician–patient communication is difficult to change [30]; however, implementing an approach that holds OCPs and patients accountable may be effective. This further solidifies the need to implement these clinical conversations surrounding PA.

Finding ways for patients to consider PA as part of their medical care is an important cultural shift. The idea of small-yet-effective encounters has also been highlighted as one way to elicit behaviour change [31,32]. Providing patients with a question prompt list offers an inexpensive way to engage patients in their care and could provide opportunities for patients to reflect on their own PA habits and ask clinicians how and what they should carry out for PA throughout their diagnosis. More education needs to be conducted on what other types of triggers may be used in clinical settings (i.e., outside programs, prompt lists, posters in waiting rooms, chart reminders). In addition, for providers, there needs to be more education in terms of how non-PA champion providers can pick up on key words (i.e., fatigue) to prompt a discussion surrounding PA.

### 4.2. Social Opportunity

The findings from this study indicate that patients living with a GB diagnosis are open to PA discussions and that they appreciate it when OCPs give them the green light to continue to engage in PA. Patients have a high amount of trust in their OCPs, and this could be due to multiple factors, including the role of OCPs in care, social norms, and the vulnerability of patients in these settings. The OCPs in the current study noted having conversations surrounding PA with patients, and they did offer PA advice at different timepoints dependent on the diagnosis and progression of the disease. A survey of 971 oncology clinicians indicated that 78.9% of respondents agreed that oncology clinicians should recommend PA to their patients [33], and another study showed that greater than 80% of patients were interested in hearing about PA from their care team [34]. There are many barriers to clinicians engaging in meaningful conversations with patients. In a study conducted with emergency room clinicians within Nova Scotia, they stated two key barriers to prescribing PA: (1) a lack of PA knowledge and (2) a need for more community-based PA programmes run by qualified exercise professionals [35] However, these barriers were not reported with GB providers. This could be due to available research exercise programs [36] and prior work with the local cancer care programme to increase knowledge of OCPs.

### 4.3. Physical Opportunity

This study shows that a barrier to PA discussion is the high workload on core care teams (i.e., nurses and physicians). In turn, this leads to a loss of supportive services for patients, as the current care team is focused on medical aspects of care. An additional barrier to implementing supportive services is clinician burnout [37], in which OCPs find it difficult to keep up with implementation efforts; this has been further enhanced by the COVID-19 pandemic [38]. Offering OCPs ways to have brief conversations and then refer to outside sources could, in turn, reduce burnout. If OCPs focus on having conversations and then refer to other team members, the core team workload and burnout could be reduced. This multidisciplinary team could result in better outcomes for patients and further enhance their care experience.

The present study shows that PA conversations can also act as a way for patients to be able to take a break from intense conversations about their diagnosis, treatment, and prognosis. Conversations surrounding prognosis and disease at the forefront of care are important; however, some OCPs may still feel uncomfortable having these palliative conversations [39]. A strategy for this could be to work on breaking up these conversations and noticing if a patient is feeling particularly distressed to open up a discussion about PA as being something that they have control over throughout treatment. As seen in the present study, many participants were encouraged by OCPs to partake in an exercise programme, and from these programs, they were able to feel like they have more control over their lives. Serious Illness Conversation Guides [40] are often used to assist OCPs to have these difficult conversations, and the further addition of a break in conversations with discussion around PA may be beneficial to both OCPs and patients. When implementing something like this, it is important to ensure that not only is the physical opportunity there for OCPs to carry out this behaviour but that the support around this discussion is also there (e.g., referral pathway).

### 4.4. Reflective Motivation

The findings from this study indicate that health decision makers are eager to take lessons from previous programs to better incorporate PA into routine care as well as lessons from what has worked in the past within Nova Scotia (i.e., paramedic programmes and smoking cessation programmes). Additionally, we need to understand how specific exercise programs have been implemented and sustained within the standard of care (i.e., cardiac rehabilitation). Health decision makers are conscious and motivated by their beliefs that PA is good for patients. The most common implementation barrier in the exercise oncology literature is organisational context [41], with this most frequently being a lack of time to discuss the aforementioned exercise programs [33]. The health decision makers from the present study understand this and are aware of the lack of time given that OCPs are pulled in many different directions. Further, OCPs can feel discouraged when they do not think patients will follow advice, and they make judgments on who will be receptive to advice, and in the current Nova Scotia Cancer Care system, there is a lack of diversity in exercise programs with little targeted outreach to equity-seeking groups [42,43,44]. This study indicates that even for patients who have not been active in the past, they feel comfortable having conversations about PA in a clinical setting. Further, those that have not been active in the past have still felt a lot of relief when partaking in PA programs that were recommended to them by their OCPs. There needs to be an understanding of how to best support these patients throughout their care journey to maintain or increase their PA levels. Further, those who face more structural barriers to participating in PA may potentially need a more personalised approach to PA counselling [45]. This study that indicates health decision makers see and understand that OCPs can and do have the reflective motivation to breach the subject of PA and that patients have a positive response to this and are motivated to listen to their OCP and change their PA behaviour.

### 4.5. Strengths and Limitations

This study is not without its limitations. These include the following: (1) the sample size was relatively small; however, it reflects a small team of experts who care for GB patients; (2) the sole interviewer at the time was heavily connected with multiple cancer and exercise studies; therefore, there could be an increased likeliness that the participants preferred PA; and (3) there may be a bias towards PA for patient and family/friend caregiver respondents. The study team tried its best to reach those that did not partake in the local GB exercise programme study [39]. However, some participants and family/friend caregivers did take part in it (2/7), and therefore, they may have had a history of positive experience with PA. Context is important, and the primary medical oncologist for GB patients in Nova Scotia is a big supporter of PA and is a co-investigator on a randomised control trial for GB patients to engage in a formal exercise programme concurrently with radiation [36]. Having a medical oncologist who is an advocate of PA may reduce the transferability of these findings to other cancers within Nova Scotia or other clinics in Canada and globally.

There are the following important strengths within this study: (1) The OCPs were well engaged in this research, and they were eager to be interviewed and express their viewpoints about this topic. Further, (2) the use of both the BCW incorporating the TDF model and the TDF domains allowed for a pointed approach to identify and work with the target behaviour and create meaningful and applicable interventions, and this approach will therefore be used in future co-design to increase the likeliness of PA discussions in clinical care.

## 5. Conclusions

It is well known that PA has a positive influence on people living with and beyond cancer [4,11,14,15,20]. This study reflects the use of PA discussions in clinical care, and the findings show that clinicians involved in care for GB patients have the knowledge to partake in PA conversations and that GB patients are open to having these conversations. From a health decision maker perspective, when designing an intervention, it is also critical to (1) not overburden clinicians and (2) make sure patients feel empowered to have meaningful conversations with clinicians. The findings highlight an ongoing need to explore means to continue to support and foster PA discussions in clinical cancer care and further formalise PA as a supportive service.

## Figures and Tables

**Table 1:** An overview of themes from the present study overlayed onto the BCW, incorporating the COM-B model and the TDF.

	Psychological Capability		Social Opportunity	Physical Opportunity		Reflective Motivation
	Cognitive and Interpersonal Skills	Memory, Attention, and Decision Processes	Social Influences	Environmental Context and Resources	Beliefs About Capabilities	Optimism
Patients/family friend caregivers	Appreciated hearing about PA from their OCPs, from initial diagnosis into follow-up appointments.		Appreciated hearing about PA from their OCPs, from initial diagnosis into follow-up appointments.	PA can aid as a break from cancer/medically focused care.	Historical PA behaviours did not mean patients were more or less likely to be open about PA discussions.	
OCPs	Use of different methods to talk about PA and offer advice that they feel patients could handle.	Formal exercise programs may act as a prompt to have a discussion around PA.				
Health system decision makers				There is a lot of information to relay to patients during appointments, and there is a need to streamline processes.		Past programme success can aid in implementation of other programs or models of care.

## Data Availability

The data presented in this study are available on request from the corresponding author due to privacy concerns.

## Supplementary Materials

The following supporting information can be downloaded at: https://www.mdpi.com/article/10.3390/curroncol32040197/s1. File S1: Interview Guides for all Sub-groups.

## Appendix A

**BCW Domain**	**TDF Domain**	**Definition**	**Examples**
**Capability**			
**Psychological capability** Knowledge or psychological skills, strength, or stamina to engage in the necessary mental processes A:D	Knowledge	Knowledge of physical activity effects on those living with non-curative cancer	Importance of physical activity counselling and education for individuals living with non-curative cancer and their family/friend caregivers
	Cognitive and interpersonal skills	Knowledge and communication skills required to provide physical activity education and support	Ability to communicate with patients regarding benefits of physical activity
	Memory, attention, and decision processes	The ability to retain knowledge about physical activity education, support, and how to access programs	Knowing about physical activity guidelines Misinformation, lack of awareness, and inconsistent recommendation among healthcare providers—they are not on the same page
	Behavioural regulation	Anything aimed at managing or changing objectively observed measured actions (such as physical activity choices)	Knowledge, skills, and action required for informed care and informed decision-making related to physical activity
**Physical capability** Physical skill, strength, or stamina	Physical skills	Ability to perform tasks related to physical activity implementation that include physical skills as well as energy/stamina to promote and supporting physical activity behaviours	Physical skills to support physical activity in cancer care
**Opportunity**			
**Social opportunity** Opportunity afforded by interpersonal influences, social cues, and cultural norms that influence the way that we think about change	Social influences	Factors within the social environment that impact discussion around physical activity, including health care provider and patient relationships, patient’s family dynamic, collaboration between health care professionals, and social/cultural norms related to physical activity	Interdisciplinary healthcare provider collaboration, physician buy-in, and leadership Community support for physical activity opportunities for positive social influences (peer support, caregiver support, education) Social acceptance of physical activity as part of cancer care
**Physical opportunity** Opportunity afforded by the environment involving time, resources, locations, cues, and physical ‘affordance’	Environmental context and resources	Any factors in the environment that encourage physical activity, including programming, resources, and technology	Physical infrastructure to be physically active in community In-person and online programming/counselling available for patients
**Motivation**			
**Automatic motivation** Automatic processes involving emotional reactions, desires (wants and needs), impulses, inhibitions, drive states, and reflex responses	Reinforcement	Increasing the probability of partaking in physical activity by arranging a dependent relationship and contingences	Convenience of physical activity to improve quality of life and maintain function Negative (or positive) experience with physical activity in the past
	Emotions	A complex reaction pattern involving experiential, behavioural, and physiological elements by which the individual attempts to deal with a personally significant matter or event	Internal healthcare provider drive to help patients and meet their needs Feelings of being let down or failure when physical activity does not go as planned
**Reflective motivation** Reflective processes involving plans (self-conscience intentions) and evaluations (beliefs about what is good and bad)	Intentions	A conscious decision to perform a behaviour (i.e., physical activity, referring to PA resources) or a resolve to act in a certain way	Healthcare provider decision to promote physical activity in practice Desire to support patient to be healthy by being physically active
	Goals	Identification of goals and responsibilities attached to their role	Want to be physically active because it is what is “best” Want to provide best possible care for patients, identifying PA as part of achieving that
	Beliefs about consequences	Acceptance of the truth, reality, or validity about outcomes of a behavioural change (e.g., physical activity implementation)	Physical activity promotes positive health outcomes Worries about not able to be physically active, fear of being judged
	Beliefs about capabilities	Acceptance of the truth, reality of validity about an ability, talent, or facility that a person can put to constructive use	Healthcare providers feeling confident that they have the experience to support physical activity Patient confidence to be physically active
	Social/professional role identity	A coherent set of behaviours and displayed personal qualities of an individual in a social and work setting	Healthcare provider advocacy for physical activity as part of practice Growing up in an environment where everyone was physically active
	Optimism	The confidence that things will happen for the best or that desired goals will be attained	Belief that substantial PA progress has happened or will happen in the future Trust and confidence that healthcare provider will support physical activity
