# Supplementary material for: How Can We Engage Oncology Care Providers and Glioblastoma Patients in Conversations About Physical Activity: A Qualitative Descriptive Study Using the Theoretical Domains Framework
Source: Curr Oncol. 2025 Mar 27;32(4):197. doi: 10.3390/curroncol32040197 (PMC12026007; doi:10.3390/curroncol32040197)
Supplement: Supplementary file 1 [file curroncol-32-00197-s001.zip › curroncol-3431443-supplementary.pdf]

Interview Guides for all Sub-groups

Semi-Structured Interview for Patients

SECTION 1: OPENING
[Introducing the Study]

As you may know, our objective in this interview is to understand the ways in which you look to view quality of life throughout your non-curative cancer diagnosis. This includes looking at challenges to ways that you seek out programs in particular ways in which you use physical activity in your life, we are looking at quality of life programs in both formal and informal ways (i.e. going to a support group or gardening). I understand that topics like this can be sensitive and first off want to thank you for taking the time to do this. I also want to say that you can stop this interview at anytime and if you need any additional supports I can provide those for you.

Do you have any questions?

If at any time you feel that the questions are too sensitive, we would be happy to turn off the recorder during that portion of the interview. You may also skip any questions you wish during the interview. If this does happen, I will write down some key notes discussed but will not attach it to your interview. As well, if you get tired throughout the interview and wish to stop we can do so and pick up at a later time.

Are you comfortable with conducting the interview now? [If no, schedule an alternative time. If yes, continue. Consent process should have already been done]
I'm going to start the recorder now.

Demographic Questions *(Will be asked open-ended but will give the following options if they do not know what the question is asking.)*

Please remember that you can choose not to answer any of the following questions.
What is your sex?

- Cisfemale
- Cismale
- Transfemale
- Transmale
- Intersex
- Prefer not to disclose

What gender do you currently identify with?

- Woman
- Man
- Agender
- Genderqueer or gender fluid
- Non- binary
- Questioning or unsure
- Other
- Prefer not to disclose

What is your martial status?

- Never married
- Married
- Common law
- Separated
- Widowed

- Divorced
- Prefer not to disclose

Education (highest level attained)

- Some High School
- Completed High School
- Some University/ College
- Completed University/ College
- Some Graduate School
- Completed Graduate School
- Prefer not to disclose

Annual Family Income

- < $20,000
- Between $20, 000- $39, 999
- Between $40, 000- $59, 999
- Between $60, 000- $79, 999
- Between 80, 000- $99, 999
- > $100, 000
- Prefer not to disclose

Current Employment Status

- Disability
- Retired
- Part time
- Homemaker
- Full Time
- Temporarily Unemployed
- Prefer not to disclose

Ethnic origin or Ancestry (Select all the apply)

- British
- Western European
- Eastern European
- Northern European
- Southern European
- Aboriginal
- East and Southern Asia
- Southern Asia
- Western Asia
- Pacific Islands
- Arab
- Latin/ Central and South American
- Caribbean
- African
- Other
- Prefer not to disclose

Non-curative cancer information
Have you had more then one cancer diagnosis in your life?
Where was your original cancer diagnosis? OR What was your cancer diagnosis?
When was your first cancer diagnosis? OR When was your diagnosis?
Have you ever been told that your cancer has spread to other parts of your body? If so what parts?

What has been your most distressing symptom from your cancer treatment and cancer diagnosis?

SECTION 1: Background questions

I would like to start off with some general questions about you.

1. Can you tell me a little bit about where you grew up and what you liked to do as a child, into your teenage years and then into your life as a young adult (i.e. into sports, board games, academics etc.)
    a. Work later on in life?
    b. Family/ friend life?

SECTION 2: Health History

Now, we are going to get into a little bit about your health history and your journey to where you are now

1. Do you have any chronic conditions besides the cancer diagnosis? (i.e. high blood pressure, diabetes, osteoporosis)
    a. And when were you diagnosed with these?
    b. Do you feel like your experiences with these other chronic conditions has prepared you to take on this new diagnosis?

SECTION 3: Non-curative Cancer Diagnosis

I was wondering if you would allow us to spend a little bit of time discussing your non-curative cancer diagnosis.

1. Can you tell me a little bit about the point of first symptoms to where we are now and your path through that?
2. Has your oncologist taken over your care now? Or do you still have contact with your family physician? How has your relationship with your family doctor changed over the past little bit?
3. Do you feel like you have adequate medical supports to deal with this diagnosis?

SECTION 4: Palliative Care and Quality of life

This next set of questions is around your experience being told you are on a palliative path and what that has meant for you. We will also chat a little bit about programs you have sought out with regards to this diagnosis to maintain your quality of life throughout your palliative path.

1. Before asking these next few questions, I want to remind you that some of these questions may be distressing for you. Remember if you do not feel comfortable answering a question you do not have to. Do you mind telling me a little bit about how your oncologist approached that you are now on a palliative path?
    a. How have you been doing since you were told this?
2. Were you given any psychosocial supports to deal with this diagnosis?
    a. One- on- one counselling?
    b. Support groups?
    c. Support groups for you and your loved ones?
3. What did your healthcare team discuss with you regarding maintaining quality of life?
4. What, if any, were some of the health and community supports or services you felt were helpful to you?

Probes:

    a. What were some of the health and community supports or services you would have liked?
    b. Did you have help identifying health and community supports or services you might need?
    c. When would you have needed or wanted those services and supports to begin?

5. Do you have any suggestions for improving community-based exercise programmes and their implementation?

SECTION 5: Physical activity programmes

I will now move onto our next section on physical activity. I want to first start off with a general definition of physical activity, every person has a different viewpoint on physical activity and it means different things to different people. Typically, physical activity is an umbrella term for anything that involves moving your body, an example of physical activity would be something like walking to work, gardening or participating in exercise. It is important to understand the different between physical activity and exercise. Exercise being a sub-set of physical activity meaning purposeful physical activity, i.e. going for a 30-minute walk or partaking in a group fitness class. We want to talk more about your experience being physical active and moving your body, which may for you include formal exercise, however it may not, and that is completely fine! Does this make sense?

1. How do you view physical activity?
    a. Have you been active your entire life?
2. What sort of physical activities do you participate in?
3. What are some positive and negative things you have seen come out of being physically active?
4. Has your relationship with physical activity changed due to your diagnosis?
5. What are the barriers to being physically active now?
6. What supports do you need to be physically active since your diagnosis? How has this changed from before your diagnosis?
7. What motivates you to stay active?
    a. Do you see physical activity as a way to help maintain your quality of life?
8. What are some other things you do to help maintain quality of life? (i.e. art, spiritual etc.)

That concludes my questions. Is there anything I've missed or anything else you'd like to add? Would you like to receive a copy of the study results? If so how do you want us to send it to you? Thank you

Semi-Structured Interview for Informal Caregivers

<u>SECTION 1: OPENING</u>

[Introducing the Study]

As you may know, our objective in this interview is to understand the ways in which you and your care recipient view quality of life throughout their non-curative cancer diagnosis. This includes looking at challenges to ways that they access quality of life enhancement programs. We are looking at quality of life programs in both formal and informal ways (i.e. going to a support group or gardening). I understand that topics like this can be sensitive and first off want

to thank you for taking the time to do this. I also want to say that you can stop this interview at anytime and if you need any additional supports I can provide those for you.

Do you have any questions?

If at any time you feel that the questions are too sensitive, we would be happy to turn off the recorder during that portion of the interview. You may also skip any questions you wish during the interview. If this does happen, I will write down some key notes discussed but will not attach it to your interview. As well, if you get tired throughout the interview and wish to stop we can do so and pick up at a later time.

Are you comfortable with conducting the interview now? [If no, schedule an alternative time. If yes, continue. Consent process should have already been done]

I'm going to start the recorder now.

Demographic Questions *(Will be asked open-ended but will give the following options if they do not know what the question is asking.)*
Please remember that you can choose not to answer any of the following questions.
What is your sex?
- Cisfemale
- Cismale
- Transfemale
- Transmale
- Intersex
- Prefer not to disclose

What gender do you currently identify with?
- Woman
- Man
- Agender
- Genderqueer or gender fluid
- Non- binary
- Questioning or unsure
- Other
- Prefer not to disclose

What is your martial status?
- Never married
- Married
- Common law
- Separated
- Widowed
- Divorced
- Prefer not to disclose

Education (highest level attained)
- Some High School
- Completed High School
- Some University/ College
- Completed University/ College
- Some Graduate School
- Completed Graduate School
- Prefer not to disclose

Annual Family Income
- < $20,000

- Between $20, 000- $39, 999
- Between $40, 000- $59, 999
- Between $60, 000- $79, 999
- Between 80, 000- $99, 999
- > $100, 000
- Prefer not to disclose

Current Employment Status

- Disability
- Retired
- Part time
- Homemaker
- Full Time
- Temporarily Unemployed
- Prefer not to disclose

Ethnic origin or Ancestry (Select all the apply)

- British
- Western European
- Eastern European
- Northern European
- Southern European
- Aboriginal
- East and Southern Asia
- Southern Asia
- Western Asia
- Pacific Islands
- Arab
- Latin/ Central and South American
- Caribbean
- African
- Other
- Prefer not to disclose
-

Interview Questions

Background

I'd like to start with some general questions about your background and experiences caring for someone with a non-curative cancer

1. Could you please tell me about your experiences caring for someone who has been diagnosed with a non-curative cancer?

Probes:

   a. What is your relationship to that individual?
   b. What was the situation you lived through with that individual (e.g. diagnosis of the individual, other family or friend supports, level of care provided to the individual)?

2. What, if any, were some of the health and community supports or services you felt were helpful to you and the person you are caring for?

Probes:

a. What were some of the health and community supports or services you would have liked?
b. Did you have help identifying health and community supports or services you might need?
c. When would you have needed or wanted those services and supports to begin?

SECTION 2: Physical activity programmes

I will now move onto our next section on physical activity. I want to first start off with a general definition of physical activity, every person has a different viewpoint on physical activity and it means different things to different people. Typically, physical activity is an umbrella term for anything that involves moving your body, an example of physical activity would be something like walking to work, gardening or participating in exercise. It is important to understand the different between physical activity and exercise. Exercise being a sub-set of physical activity meaning purposeful physical activity, i.e. going for a 30-minute walk or partaking in a group fitness class. We want to talk more about you and your care recipients experience being physically active and moving your body, which may for you include formal exercise, however it may not, and that is complete fine! Does this make sense?

1. How do you view physical activity?
    a. Have you been active your entire life?
2. What sort of physical activities do you participate in?
    a. Do you participate in any physical activity with your care recipient?
    b. How has your relationship with physical activity changed over the course of their diagnosis?
3. Has your relationship with physical activity changed due to their diagnosis?
    a. Do you feel like you lack time to be physically active if you are going to a lot of healthcare appointments with your care recipient?
4. Are you and your care recipient active together at all? OR do your care recipient partake in physical activity on their own?
    a. What are some positive and negative things you have seen come out of your care recipient being physically active?
5. What supports do you and your partner need to be physically active after their diagnosis? How has this changed from before their diagnosis?
6. What motivates you to stay active?
    a. Do you see physical activity as a way to help maintain your quality of life? What about your care recipients?
7. What are some other things you and your care recipient do to help maintain quality of life? (i.e. art, spiritual etc.)

8. Do you have any suggestions for improving community-based exercise programmes and its implementation? These could either be for yourself, your care recipient or something you can do together.

That concludes my questions. Is there anything I've missed or anything else you'd like to add? Would you like to receive a copy of the study results? If so how do you want us to send it to you? Thank you

Semi-Structured Interview for Healthcare providers

<u>SECTION 1: OPENING</u>

[Introducing the Study]

As you may know, our objective in this interview is to understand the ways in which individuals with non-curative cancer partake in programs to maintain or slow the regression of their quality of life throughout their diagnosis. This includes looking at barriers and facilitators to ways that they access quality of life enhancement programs, we are looking at quality of life programs (specifically related to physical activity) in both formal and informal ways (i.e. going to a support group or gardening). We want to know your experiences with your patients and how you discuss quality if life with them and what sort of resources your provide to your patient, these can be formal or informal.

Do you have any questions?

If at any time you feel that the questions are too sensitive, we would be happy to turn off the recorder during that portion of the interview. You may also skip any questions you wish during the interview. If this does happen, I will write down some key notes discussed but will not attach it to your interview. As well, if you get tired throughout the interview and wish to stop we can do so and pick up at a later time.

Are you comfortable with conducting the interview now? [If no, schedule an alternative time. If yes, continue. Consent process should have already been done]
I'm going to start the recorder now.

Demographic Questions *(Will be asked open-ended but will give the following options if they do not know what the question is asking.)*
Please remember that you can choose not to answer any of the following questions.
What is your sex?

- Cisfemale
- Cismale
- Transfemale
- Transmale
- Intersex
- Prefer not to disclose

What gender do you currently identify with?

- Woman
- Man
- Agender
- Genderqueer or gender fluid
- Non- binary
- Questioning or unsure
- Other
- Prefer not to disclose

Ethnic origin or Ancestry (Select all the apply)

- British
- Western European
- Eastern European
- Northern European
- Southern European
- Aboriginal
- East and Southern Asia
- Southern Asia
- Western Asia
- Pacific Islands
- Arab

- Latin/ Central and South American
- Caribbean
- African
- Other
- Prefer not to disclose
-

I'd like to start with some general questions about your position and responsibilities.

1. What is your position in the Health Authority or the Department of Health and Wellness?
    a. To whom do you report (position/title, not name)?
2. How long have you been working there?
    a. How long working in this position?
    b. What are your main responsibilities?
3. Why did you choose to be involved with GBM patients?

I now want am hoping to learn more about your work with patients living with non-curative cancer and ways in which you refer to quality of life enhancing programs and how you view quality of life in these patients as a whole

1. What are current resources you either suggest to your patients or directly refer to your patients (i.e. support groups, spiritual-based programmes, caregiver programmes, palliative care services)
    a. How do you find these help your patients?
    b. Do you differ what programmes your offer based on their goals of care?
2. Do you find there are too little or too many resources for patients?
    a. Why do you think this way?
3. Do you have a process to check back in with patients about programmes you offer or refer them to?

I now want to start talking about ways in which the NS or CB Cancer Center work with new initiatives and how they are implemented into practice.

1. How has your place of work dealt with implementing new types of care or new initiatives? Do you have any examples?
    a. What drove these new practices to be implemented?
    b. How have any new practices changed the way you view your work and the type of care you give?
2. What has been the greatest way to enhance uptake of new practices in your cancer center? (i.e. champions, incentives, etc.)
    a. Biggest challenge?
3. How have you personally been involved in implementation of programmes in the past?
    a. Do you have any future asks for programming in the future?

My next set of questions is specifically aimed towards physical activity as a way to maintain, slow the regression or improve quality of life for those living with a non-curative cancer. I want to first start off with saying what physical activity is, every person has a different viewpoint on physical activity and it means different things to different people. Typically, physical activity is an umbrella term for anything that involves moving your body, an example of physical activity would be something like walking to work, gardening or participating in exercise. It is important to understand the difference between physical activity and exercise. Exercise being a sub-set of physical activity meaning purposeful physical activity, i.e. going for a 30-minute walk or partaking in a group fitness class. We want to talk more about your experience in

referring or prescribing physical active, which may include formal exercise, however it may not, and that is completely fine! Does this make sense?

1. How do you view physical activity?
    a. Do you refer or prescribe physical activity to your patients?
    b. Do you yourself participate in physical activity?
2. Do you mention physical activity in your appointments with your patients?
    a. If so how often and is it formally or informally?
3. What are some positive and negative things you have seen come out of your patients being physically active? If they have disclosed this to you.
4. What are the barriers to being prescribing or referring to physical activity programmes?
5. What could be done to promote and sustain physical activity in healthcare?
6. Do you feel well-informed on the benefits of physical activity for your patients?
    a. In what ways do you think you would feel more comfortable discussing physical activity with your patients? Are there any resources that could help you with this?
7. What are other ways you work with your patient to enhance and/or maintain their quality of life?

That concludes my questions. Is there anything I've missed or anything else you'd like to add? Would you like to receive a copy of the study results? If so how do you want us to send it to you? Thank you

Semi-Structured Interview for Health Decision Makers

<u>SECTION 1: OPENING</u>
[Introducing the Study]

As you may know, our objective in this interview is to understand the ways in which individuals with non-curative cancers partake in programs to maintain or slow the regression of their quality of life throughout their diagnosis. This includes looking at barriers and facilitators to ways that they access quality of life enhancement programs, we are looking at quality of life programs in both formal and informal ways (i.e. going to a support group or gardening). We want to know your experiences with implementation of initiatives into the cancer center, and how previous programmes have

Do you have any questions?

If at any time you feel that the questions are too sensitive, we would be happy to turn off the recorder during that portion of the interview. You may also skip any questions you wish during the interview. If this does happen, I will write down some key notes discussed but will not attach it to your interview. As well, if you get tired throughout the interview and wish to stop we can do so and pick up at a later time.

Are you comfortable with conducting the interview now? [If no, schedule an alternative time. If yes, continue. Consent process should have already been done]

I'm going to start the recorder now.

Demographic Questions *(Will be asked open-ended but will give the following options if they do not know what the question is asking.)*
Please remember that you can choose not to answer any of the following questions.

What is your sex?

- o Cisfemale
- o Cismale
- o Transfemale
- o Transmale
- o Intersex
- o Prefer not to disclose

What gender do you currently identify with?

- o Woman
- o Man
- o Agender
- o Genderqueer or gender fluid
- o Non- binary
- o Questioning or unsure
- o Other
- o Prefer not to disclose

Ethnic origin or Ancestry (Select all the apply)

- o British
- o Western European
- o Eastern European
- o Northern European
- o Southern European
- o Aboriginal
- o East and Southern Asia
- o Southern Asia
- o Western Asia
- o Pacific Islands
- o Arab
- o Latin/ Central and South American
- o Caribbean
- o African
- o Other
- o Prefer not to disclose

I'd like to start with some general questions about your position and responsibilities.

4. What is your position in the Health Authority or the Department of Health and Wellness?
    a. To whom do you report (position/title, not name)?
5. How long have you been working there?
    a. How long working in this position?
    b. What are your main responsibilities?
6. Why did you lean more towards your position as opposed to other path?

I now want to start talking about ways in which the NS or CB Cancer Center work with new initiatives and how they are implemented into practice.

4. How has your place of work dealt with implementing new practices? Do you have any examples?
    a. What drove these new practices to be implemented?
    b. How have any new practices changed the way you view your work and the type of care you give?
5. What has been the greatest way to enhance uptake of new practices (i.e. new care considerations, new EMR systems, new types of care) in your cancer center? (i.e. champions, incentives, etc.)

a. Biggest challenge?

6. How have you personally been involved in implementation of programmes in the past?
    a. Do you have any future asks for programming in the future?

My next set of questions is specifically aimed towards physical activity as a way to maintain, slow the regression or improve quality of life for those living with a non-curative cancer. I want to first start off with saying what physical activity is, every person has a different viewpoint on physical activity and it means different things to different people. Typically, physical activity is an umbrella term for anything that involves moving your body, an example of physical activity would be something like walking to work, gardening or participating in exercise. It is important to understand the difference between physical activity and exercise. Exercise being a sub-set of physical activity meaning purposeful physical activity, i.e. going for a 30-minute walk or partaking in a group fitness class. We want to talk more about your experience in referring or prescribing physical active, which may include formal exercise, however it may not, and that is completely fine! Does this make sense?

8. How do you view physical activity?
    a. Do you yourself participate in physical activity?
9. Has there been any history of physical activity programme implementation in your cancer center that you are aware of?
10. What are the barriers you see to implementing physical activity programmes formally within the cancer center?
11. What could be done to promote and sustain physical activity in healthcare?
12. Do you feel well-informed on the benefits of physical activity for the general population? As well as for those that are diagnosed with a non-curative cancer?
13. What are other programmes that you are aware of to help patients maintain/ improve quality y of life throughout their diagnosis?
    a. Are there any programmes that are on "the fringe" and to be implemented?

That concludes my questions. Is there anything I've missed or anything else you'd like to add? Would you like to receive a copy of the study results? If so how do you want us to send it to you? Thank you
